# A comprehensive review on microbial production of 1,2-propanediol: micro-organisms, metabolic pathways, and metabolic engineering

**DOI:** 10.1186/s13068-021-02067-w

**Published:** 2021-11-18

**Authors:** Yuan-ming Tao, Chong-yang Bu, Li-hua Zou, Yue-li Hu, Zhao-Juan Zheng, Jia Ouyang

**Affiliations:** 1 Jiangsu Co-Innovation Center of Efficient Processing and Utilization of Forest Resources, Nanjing Forestry University, Nanjing, 210037 People’s Republic of China; 2 College of Chemical Engineering, Nanjing Forestry University, Nanjing, 210037 People’s Republic of China

**Keywords:** 1,2-Propanediol, Micro-organism, Metabolic pathway, Metabolic engineering

## Abstract

1,2-Propanediol is an important building block as a component used in the manufacture of unsaturated polyester resin, antifreeze, biofuel, nonionic detergent, etc. Commercial production of 1,2-propanediol through microbial biosynthesis is limited by low efficiency, and chemical production of 1,2-propanediol requires petrochemically derived routes involving wasteful power consumption and high pollution emissions. With the development of various strategies based on metabolic engineering, a series of obstacles are expected to be overcome. This review provides an extensive overview of the progress in the microbial production of 1,2-propanediol, particularly the different micro-organisms used for 1,2-propanediol biosynthesis and microbial production pathways. In addition, outstanding challenges associated with microbial biosynthesis and feasible metabolic engineering strategies, as well as perspectives on the future microbial production of 1,2-propanediol, are discussed.

## Background

The traditional petrochemical industry needs further reforms due to public concerns over the pollution of the environment and the shortage of petroleum resources [1, 2]. However, because of the wider range of biomass resources, safer manufacturing processes and lower effects on the environment today, the bio-based chemical industry is becoming increasingly powerful in the chemical manufacturing arena [3]. Currently, the increasing production of chemicals from biomass via biotechnological routes has captured the attention of researchers, and these chemicals include biofuels (ethanol, butanol) [4, 5], pharmaceuticals (vitamins) [6], organic acids (lactic acid and succinic acid) [7, 8], diols (1,2-propanediol, 1,3-propanediol) [9, 10] and other platform bulk and specialty chemicals.

1,2-Propanediol (1,2-PDO), as a C3 diol, is an important platform chemical with high demand in industry [9]. To date, 1,2-PDO has been widely used in the building material, chemical and pharmaceutical industries as a monomer for use in producing polyester resins, antifreeze agents, liquid detergents, biofuels, cosmetics, food, etc. [11–15] Annually, more than 1.36 million tons of merely racemic 1,2-PDO is produced due to global demand and reached approximately $0.373 billion globally in 2020, and it is expected to reach more than $0.398 billion by 2026 with a CAGR (Compound Annual Growth Rate) of 1.6%. Currently, the commercial route to 1,2-PDO involves the hydration of fossil fuel-based propylene via chemical methods [16]. The use of fossil resources as the initial raw material in these methods not only pollutes the environment but also results in a racemic mixture [17]. In addition, two stereoisomers exist in 1,2-PDO: *R*-1,2-PDO and *S*-1,2-PDO. Compared with racemic products, the pure stereoisomers of this chemical demonstrate greater potential as chiral synthons in the organic synthesis of chiral pharmaceutical products. Nevertheless, the application of these pure stereoisomers is often restricted due to their high price and low availability, except at the laboratory scale [18]. For these reasons, special concern has emerged with respect to the production of 1,2-PDO, especially in a pure stereoisomer form from biomass via biological processes.

Although the production of 1,2-PDO by a variety of bacteria and yeasts has been achieved for many years, the biological process is still highly challenging due to the lack of naturally efficient synthetic pathways. For example, microbial 1,2-PDO production has not been applied to industrial scale manufacturing because of low yield. However, in recent years, along with the rapid development in metabolic strategies, including modification of natural pathways and design of artificial pathways, the production of a pure stereoisomer of 1,2-PDO from inexpensive substrates via biological routes for industrial applications is currently possible. Herein, recent efforts on strain exploration, process optimization, pathway designation and various metabolic engineering strategies to improve microbial production of 1,2-PDO are summarized. Furthermore, the drawbacks, challenges, and future trends towards economical manufacturing of 1,2-PDO via biotechnological routes are discussed.

## Discovery of natural micro-organisms with the capability to produce 1,2-propanediol

A variety of micro-organisms have been reported to have the capability of producing 1,2-PDO in nature, including bacterial strains of the genera *Prevotella* [19], *Salmonella* [20], *Klebsiella* [20], *Clostridium* [21–23] and *Lentilactobacillus* [24]; fungal strains of the genera *Yamadazyma* and *Debaryomyces* [25]; and several *Saccharomyces* species [26]. However, for different types of micro-organisms, the substrates and fermentation conditions used and the reaction mechanisms vary greatly. For example, 1,2-PDO production by bacteria requires strictly anaerobic conditions, the direct opposite of that needed for production by fungi. Metabolism of different substrates leads to the stereochemistry of 1,2-PDO produced naturally by bacteria; fucose and rhamnose can generate *S*-1,2-PDO, but glucose and xylose can generate the *R*-isomer.

Among the reported microbial genera with 1,2-PDO production capacity, most have been confirmed to produce 1,2-PDO mainly through the use of various sugars, such as fucose, rhamnose, and glucose. The formation of 1,2-PDO was first reported as a product of cellulose decomposition in *Clostridium thermobutyricum*. Thereafter, many bacteria were found to produce *S*-1,2-PDO from fucose or rhamnose [27]. As shown in Table 1, in an early report, Turner et al. suggested that *Bacteroides ruminicola* has the capability to utilize l-rhamnose for naturally producing 1,2-PDO. Approximately 0.92 mol of 1,2-PDO was detected per mol of rhamnose, while 0.36 mol acetate, 0.02 mol formate and 0.29 mol succinate were synchronously produced [19]. Subsequently, the fermentation mechanism of fucose and rhamnose in *Salmonella typhimurium* and *Klebsiella pneumonia* was investigated [20]. It was found that both of these species excreted 1,2-PDO when grown merely anaerobically with fucose or rhamnose. This phenomenon was explained that a propanediol oxidoreductase critical for the reduction of lactaldehyde to 1,2-PDO was induced in *S. typhimurium* in an anaerobic environment, and the presence of oxygen possibly prevented the enzymatic activity. During this process, the production of 1,2-PDO was a result of an attempt to regenerate oxidized NAD. Anaerobic conditions seemed to be important for the production of 1,2-PDO when fucose or rhamnose was the sole source of carbon. More recently, a thermophilic anaerobe, *Clostridium* strain AK-1, was found to produce *S*-1,2-PDO from l-rhamnose. Approximately 22.13 mM 1,2-PDO was produced with a maximum yield of 0.81 mol 1,2-PDO/mol from l-rhamnose [22].

**Table 1:** Microbial production of 1,2-propanediol using natural strains

Organisms	Substrates	Fermentation modes	Titer (g/L)	Yield (g/g)	References
Bacteria					
*Bacteroides ruminicola*	l-Rhamnose	Batch	0.070	0.0853	[19]
*Salmonella typhimurium*	l-Fucose	Flask	1.37	0.417	[20]
*Salmonella typhimurium*	l-Rhamnose	Flask	1.46	0.445	[20]
*Klebsiella pneumoniae*	l-Fucose	Flask	1.49	0.454	[20]
*Klebsiella pneumoniae*	l-Rhamnose	Flask	1.52	0.463	[20]
*Clostridium* strain AK-1	l-Rhamnose	Batch	1.68	0.375	[22]
*Clostridium thermosaccharolyticum* HG-8	Glucose	Batch	9.05	0.20	[21]
*Clostridium thermosaccharolyticum* HG-8	Glucose	Fed-batch	7.90	0.27	[23]
*Clostridium thermosaccharolyticum* HG-8	Xylose	Flask	3.23	0.123	[23]
*Clostridium thermosaccharolyticum* HG-8	Mannose	Flask	3.1	0.115	[23]
*Clostridium thermosaccharolyticum* HG-8	Cellobiose	Flask	3.1	0.108	[23]
*Clostridium thermosaccharolyticum* HG-8	Whey permeate	Flask	2.8	–	[29]
*Lactobacillus buchneri*	Lactic acid	Flask	0.571	0.384	[24]
Yeast					
*Candida polymorpha*	l-Rhamnose	Flask	–	–	[25]
*Pichia robertsii*	l-Rhamnose	Flask	–	–	[25]
*Saccharomyces cerevisiae*	Citrus stillage	Flask	0.211	–	[26]
*Saccharomyces cerevisiae*	Whey stillage	Flask	0.123	–	[26]
*Saccharomyces cerevisiae*	Corn stillage	Flask	0.105	–	[26]

As mentioned above, these studies confirmed the microbial production of *S*-1,2-PDO from rhamnose or fucose under anaerobic conditions. However, the route of this production is not commercially feasible due to the high cost of the substrate and low level of production [17]. Hence, a search for 1,2-PDO production from inexpensive, readily available sugars, such as glucose, xylose and arabinose, was performed, and then these substrates were developed for 1,2-PDO production. Compared with bacteria utilizing fucose or rhamnose as a source of 1,2-PDO synthesis, *Clostridium* strains ferment glucose or xylose anaerobically for 1,2.-PDO production. Several studies found that these strains can produce enantiomerically pure *R*-1,2-PDO during this process [18, 21]. Moreover, a methylglyoxal pathway of 1,2-PDO production by *Clostridium* strains has been proposed, and methylglyoxal synthase (*mgsA*) has been found to be important for 1,2-PDO production. In *Clostridium sphenoides*, *R*-1,2-PDO was found to be produced from glucose only under phosphate limitation, as methylglyoxal synthase activity is strongly inhibited by phosphate [28]. *Clostridium thermosaccharolyticum* can produce *R*-1,2-PDO greater than 99% enantiomeric excess from many kinds of sugars, including glucose, xylose, mannose and cellobiose [23]. *R*-1,2-PDO (9.05 g/L) with the best yield of 0.20 g/g glucose was achieved from 45 g/L glucose after 25 h fermentation at 60 °C and pH 6.0 under a N_2_ atmosphere, and d-lactate was the major product of this fermentation process with 11.12 g/L [21]. To our knowledge, this is the highest level of 1,2-PDO produced using natural organisms. Later, in 2001, *C. thermosaccharolyticum* HG-8 was found to use a wider range of sugars to produce 1,2-PDO than previously reported, including lactose found in cheese whey, and d-glucose, d-galactose, l-arabinose, and d-xylose found in corn and wood byproducts; this strain afforded a maximum 1,2-PDO concentration of 2.8 g/L when hydrolysed whey permeate in yeast extract was used; in addition, it produced 7.9 g/L lactate, 3.9 g/L acetate and 2.1 g/L acetol [29]. These studies showed *C. thermosaccharolyticum* to be a suitable natural producer for enantiopure *R*-1,2-PDO due to its thermophilic fermentation properties and ability to utilize various renewable residues.

In addition to the abovementioned organisms using various different sugars as substrates for 1,2-PDO production, lactate, an inexpensive and readily available chemical obtained by fermentation, can be applied for 1,2-PDO production by *Lactobacillus buchneri* and its close relatives [24]. Due to fewer reaction steps and more accessible substrates, the production of useful chemicals from lactic acid via chemical and biotechnological routes represents the green chemistry of the future, compared to their production from various sugars. Elferink et al. found that *L. buchneri* is capable of converting lactate into equimolar amounts of acetic acid and 1,2-PDO [24]. In this study, acidic and anoxic conditions seemed to be necessary for lactate degradation by *L. buchneri*. Thus, it was proposed that its anaerobic lactate-degrading capacity needs to be induced by environmental conditions, such as pH and temperature.

Besides 1,2-PDO production by bacteria, it is also worth mentioning that there were several reports on aerobic 1,2-PDO production by some yeasts, such as *Candida polymorpha* and *Pichia robertsii*, in the late 1960s [25]. A good 1,2-PDO yield of 38% from sugar consumed was obtained, especially in *Candida polymorpha*. In addition, although a small quantity of 1,2-PDO has been detected in several industrial efforts based on *Saccharomyces cerevisiae* fermentation, the full metabolic network of 1,2-PDO production in yeast is still unclear [26]. Early phenomena suggested some basic enzymes for 1,2-PDO production are present in yeast [30]. Subsequently, the successful isolation of methylglyoxal synthase from *S. cerevisiae* clearly supported this hypothesis [31]. This enzyme was proven to be insensitive to phosphate, in contrast to *E. coli* methylglyoxal synthase, providing a promising new fermentation host for 1,2-PDO production.

## Biosynthetic pathways for the production of 1,2-propanediol and the enzymes involved

Currently, 1,2-PDO is mainly produced through chemical routes using propylene oxide from the petrochemical industry [32]. Although some efforts have been made to use biological methods to synthesize 1,2-PDO from biomass, the titres, yields and productivity of biological methods remain low, and the bioprocess is cost-ineffective. Hence, there is a great need to deeply investigate and understand these biosynthetic pathways with natural organisms to develop bioprocesses for more-efficient industrial production. As mentioned above, bacteria have multiple strategies for producing 1,2-PDO with different substrates. These pathways are summarized below. The metabolic pathways of 1,2-PDO production can be divided into three routes: the deoxyhexose pathway [33], methylglyoxal pathway [23], and lactate pathway [24]. Although these pathways involve different intermediates and enzymes, these bioprocesses are all effective under only anaerobic conditions. Among these pathways, the deoxyhexose pathway is the primary route of *S*-1,2-PDO production, and the methylglyoxal pathway is the primary route of *R*-1,2-PDO production, but the chiral status of 1,2-propanediol synthesized via the lactic acid pathway is not clear.

### Deoxyhexose pathway

**Fig. 1:**
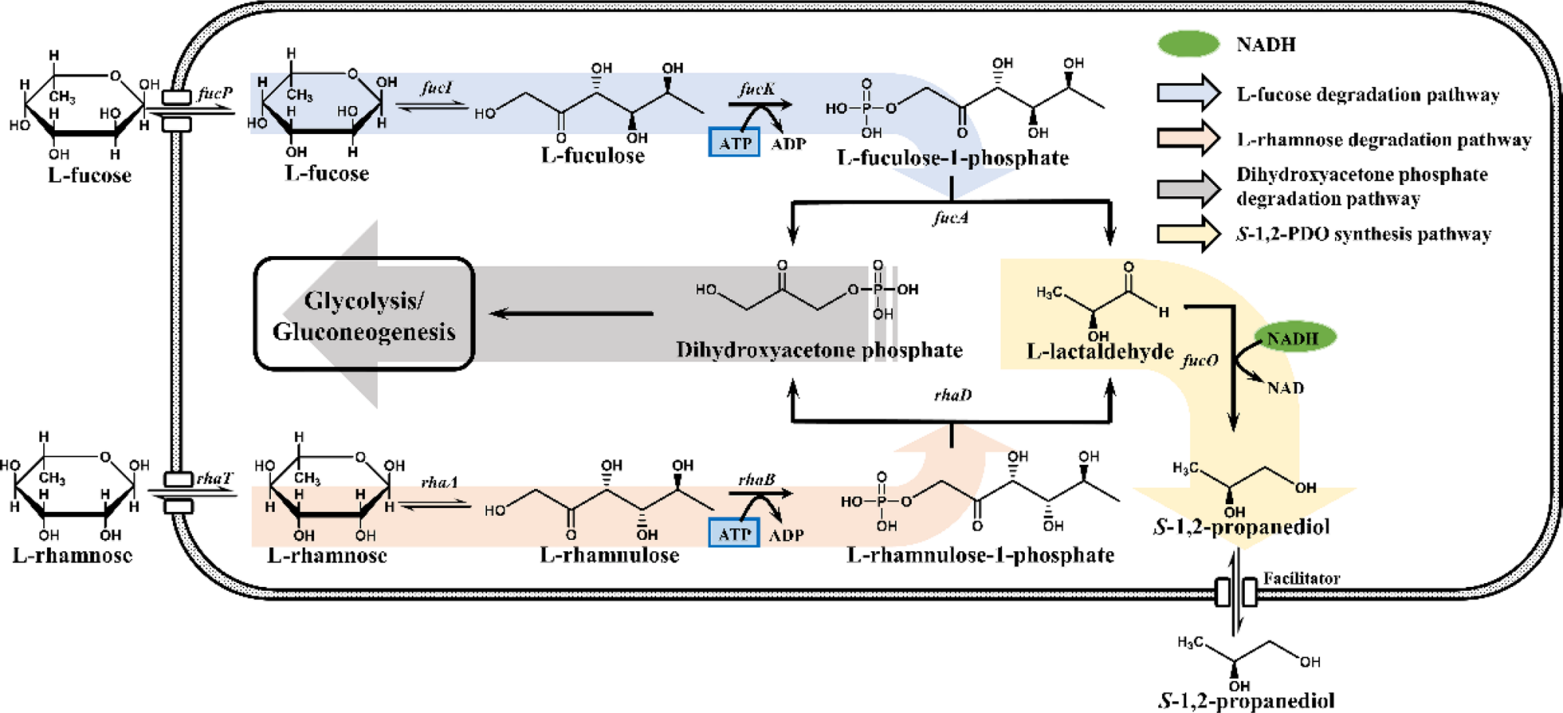
Metabolic pathways for the production of 1,2-PDO from l-fucose and l-rhamnose (Deoxyhexose pathway). The genes in Fig. 1 are all from *E. coli*. *fucP*: l-fucose permease; *rhaT*: l-rhamnose permease; *fucI*: l-fucose isomerase; *rhaA*: l-rhamnose isomerase; *fucK*: l-fuculokinase; *rhaB*: l-rhamnulokinase; *fucA*: l-fuculose-1-phosphate aldolase; *rhaD*: l-rhamnulose-1-phosphate aldolase; *fucO*: propanediol oxidoreductase

Because l-fucose and l-rhamnose are known to be catabolized in anaerobic environments, the biosynthetic pathway of 1,2-PDO production from l-fucose and l-rhamnose was first explored and identified in *Salmonella*, *Klebsiella* [20], *Clostridium* [21–23] and *Prevotella* [19]. As shown in Fig. 1, the deoxyhexose pathway consists of several steps. First, the two main deoxyhexoses are converted into l-rhamnose-1-phosphate or l-fucose-1-phosphate in the presence of isomerase and kinase, respectively, which are subsequently broken down by aldolase to l-lactaldehyde and dihydroxyacetone phosphate (DHAP). Then DHAP is converted into pyruvate through a series of reactions. NADH generated in the metabolism is consumed in the 1,2-PDO oxidoreductase-catalyzed reduction of lactaldehyde into S-1,2-PDO.

For 1,2-PDO biosynthesis from l-fucose or l-rhamnose, four key enzymes are involved, i.e., l-fucose/rhamnose isomerase (*fucI*/*rhaA*) [34, 35], l-fuculokinase/rhamnulokinase (*fucK*/*rhaB*) [36, 37], l-fuculose-1-phosphate/rhamnulose-1-phosphate aldolase (*fucA*/*rhaD*) [38, 39] and propanediol oxidoreductase (*fucO*) [40]. The isomerase, kinase, and aldolase were found to be functional in both aerobic and anaerobic environments, which means that the formation of neither DHAP nor l-lactaldehyde was influenced by the environment [41]. Upon the release of DHAP and l-lactaldehyde, DHAP participates in both gluconeogenic and glycolytic processes as an important intermediate in central metabolism. However, the fate of l-lactaldehyde is influenced by the activity of propanediol oxidoreductase [42]. Earlier reports showed that propanediol oxidoreductase exhibits almost 70% post-transcriptional inactivation in the presence of oxygen, while l-lactaldehyde is completely converted to pyruvate through two steps of the oxidation process [41]. Accordingly, *S*-1,2-PDO is obtained only from l-lactaldehyde by *fucO* under anaerobic conditions. Nevertheless, although early studies indicated that an anaerobic environment is necessary for the deoxyhexose pathway, recent research revealed that l-lactaldehyde can be reduced into *S*-1,2-PDO in aerobic environments, and for these cases, the regulatory mechanism represented by the NADH/NAD^+^ ratio and an efficient l-lactaldehyde detoxification process are recognized as possible explanations. In addition, a rather detailed intracellular flux distribution of this pathway has been identified by the stable isotope tracer technique, which is useful for obtaining profound information on the functioning of a metabolic network [43]. Although it has been extensively investigated for years, the deoxyhexose metabolic pathway is not economical for commercialization due to the high price and difficult acquisition of l-fucose and l-rhamnose; these challenges have prompted further study into the reliability of the methylglyoxal pathway.

### Methylglyoxal pathway

**Fig. 2:**
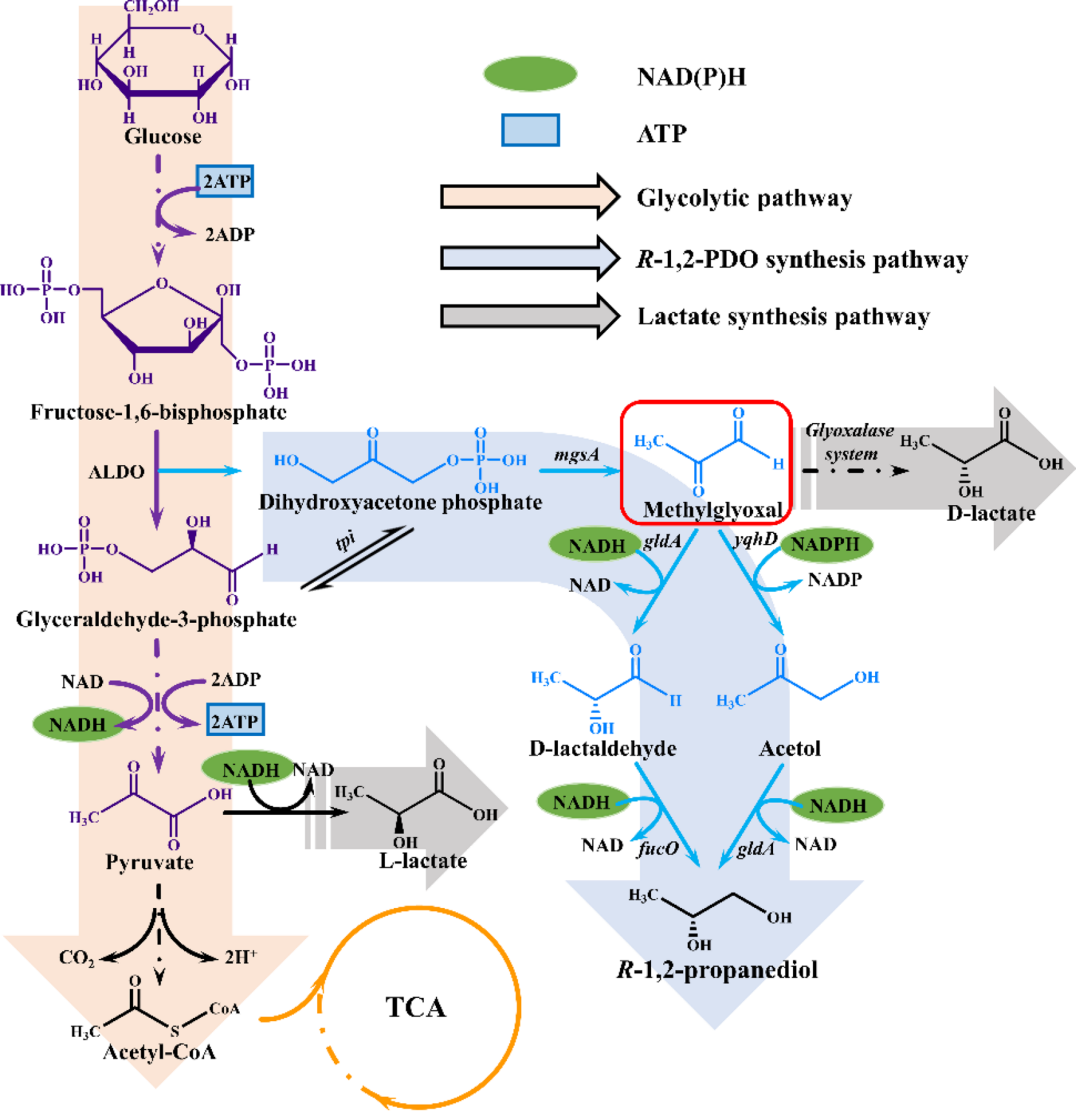
Metabolic pathways for the production of 1,2-PDO from glucose (Methylglyoxal pathway). The genes in Fig. 2 are all from *E. coli*. ALDO: fructose–bisphosphate aldolase; *tpi*: triose-phosphate isomerase; *mgsA*: methylglyoxal synthase; *gldA*: glycerol dehydrogenase; *yqhD*: alcohol dehydrogenase; *fucO*: propanediol oxidoreductase; Glyoxalase system: glyoxalase I (lactoylglutathione lyase), glyoxalase II (hydroxyacylglutathione hydrolase)

In addition to the deoxyhexose pathway, another metabolic pathway based on methylglyoxal has been reported in *Clostridium thermosaccharolyticum* and *Clostridium sphenoides*, which have been found to produce 1,2-PDO by fermenting glucose, fructose, mannose, galactose, xylose, arabinose, lactose or cellobiose [23, 28]. In this pathway, take the glucose as an example, the substrate is first converted to fructose-1,6-biphosphate, which is cleaved into DHAP and glyceraldehyde 3-phosphate (Fig. 2). Glyceraldehyde 3-phosphate is then converted to L-lactate or enters the TCA cycle. On the other hand, DHAP is converted into methylglyoxal as a key intermediate by *mgsA*. The latter is subsequently reduced to 1,2-PDO through acetol or lactaldehyde in the presence of propanediol oxidoreductase/alcohol dehydrogenase (*fucO*/*yqhD*) and then glycerol dehydrogenase (*gldA*) [23]. Methylglyoxal synthase was considered as the key enzyme in the methylglyoxal pathway. A large number of micro-organisms have been reported to possess methylglyoxal synthase activity, including *Pseudomonas saccharophila* [23], *Escherichia coli* [44], *Proteus vulgaris* [45] and several *Clostridium* [28]. As previously reported, methylglyoxal synthase is strongly inhibited by phosphate in most micro-organisms. In *Clostridium sphenoides*, *R*-1,2-PDO was synthesized only via the methylglyoxal pathway when the phosphate concentration was less than 80 μM, which was insufficient to trigger the phosphate-induced inhibitory mechanism. Nevertheless, the results of later studies on methylglyoxal synthase in *C*. *thermosaccharolyticum* HG-8 indicating the nonexistence of phosphate inhibition up to 113 mM phosphate [23]. This phenomenon was also observed in *S. cerevisiae*. Therefore, further research on the phosphate inhibitory mechanism is required for the in-depth application of the methylglyoxal pathway.

Compared to the deoxyhexose pathway, the substrates for the methylglyoxal pathway are less expensive, and *R*-1,2-PDO is mainly produced. Nevertheless, the cytotoxic effect of methylglyoxal, which can suppress protein synthesis, is not negligible [46]. Notably, methylglyoxal can take advantage of the interaction with ribosomes to suppress protein synthesis. Thus, slow cell growth and metabolic imbalance are important issues regarding 1,2-PDO production through this pathway.

### Lactate pathway

**Fig. 3:**
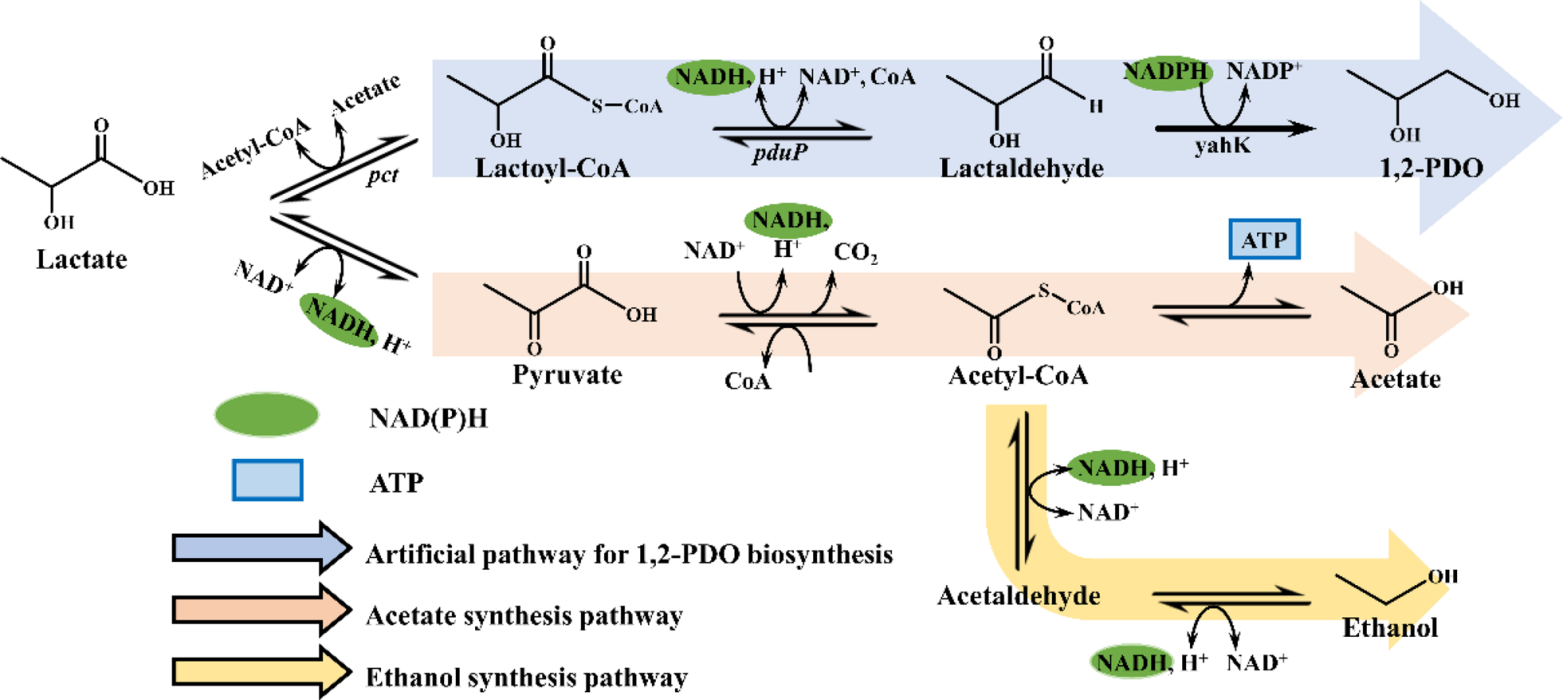
Metabolic pathways for the production of 1,2-PDO from lactate (Lactate pathway). Acetate and ethanol synthesis pathway are from the proposed pathway for anaerobic degradation of lactic acid by *L. buchneri* [24]. *Pct*: propionate CoA-transferase (*M. elsdenii*); *pduP*: propanal dehydrogenase (*S. enterica*); *yahK*: lactaldehyde reductase (*E. coli*)

Consequently, the lactate pathway, which is capable of preventing the synthesis of methylglyoxal, is a newly recognized and promising route for 1,2-PDO microbial production. In 2001, Elferink et al. reported an observation that lactic acid was reduced to 1,2-PDO under anaerobic and acidic condition. Pathway was proposed for the anaerobic degradation of lactic by the authors (Fig. 3) [24]. In this study, *Lactobacillus brucei* and *Lactobacillus parabuchneri* successfully degraded 1 mol of lactic acid into 0.5 mol of acetic acid and 0.5 mol of 1,2-PDO with the concomitant accumulation of ethanol but without an external electron acceptor. One of the explanations for these outcomes suggests that *L. buchneri* and *L. parabuchneri* eliminate excess reducing equivalents by producing 1,2-PDO. In addition, because the degradation of lactic acid is strongly affected by pH, a protective mechanism against a low-pH environment has been proposed. That is, the degradation of lactic acid into 1,2-PDO and acetic acid, which has a higher pK_a_, is thought to protect against cell destruction due to the overaccumulation of undissociated organic acids in acidic environments. In the proposed pathway, nearly one-half of the lactic acid is first reduced to lactaldehyde and then the 1,2-PDO is achieved from the lactaldehyde. The another one-half of the lactic acid is oxidized to acetate to provide the required reducing equivalents in the form of NADH, along with small amounts of ethanol that is excretion over the same time period. The proposed pathway may not produce toxic intermediate methylglyoxal and the *Lactobacillus* strains can also be cultured under acidic conditions, which provides a new example for the synthesis of 1,2-PDO by micro-organisms. However, biochemical details of the pathway are unknown.

Based on retrosynthetic analysis, an artificial pathway for biosynthesis of 1,2-PDO from glucose via the intermediacy of lactic acid was devised [47]. The lactaldehyde is formed from the lactic acid under the combined action of propionate CoA-transferase (*pct*) and propanal dehydrogenase (*pduP*) and then further reduced to 1, 2-PDO by lactaldehyde reductase (*yahK*). By overexpressing these enzymes in *E. coli*, the highest titre of 1,2-PDO was successfully synthesized (*R*-1,2-PDO was produced at 17.3 g/L, while the *S*-isomer was produced at 9.3 g/L), which shows the great potential of this reaction pathway [48].

## Metabolic engineering strategies for the enhanced production of 1,2-propandiol

Although various micro-organisms with different metabolic pathways have been identified that are available for microbial production of 1,2-PDO, there are many factors hindering the 1,2-PDO commercialization process. For example, biosynthesis of 1,2-PDO is a reduction process in which many of the reductases depend on the cofactor NAD(P)H; therefore, a process to increase the supply of redox cofactors sufficiently is urgently needed to improve product yield. In addition, in the three abovementioned pathways, 1,2-PDO is not the sole product, regardless of whether sugars or lactic acid are the substrates. This means that a low theoretical yield (< 50%) seems to be unavoidable. In addition, the accumulation of toxic intermediates is an obstacle that must be removed to increase product concentration and yield. Recently, with the advancement of genetic engineering technology, applying systematic metabolic engineering and coenzyme regulation strategies to strengthen 1,2-PDO organisms has provided feasible strategies for solving the aforementioned problems, including the enhancement of major metabolic pathways and substrate utilization range through the introduction of heterologous genes and overexpression of endogenous genes, the redistribution of carbon flux through the knockout of genes encoding by-product pathways and the improvement of the supply of cofactors through cutting off additional NAD(P)H consumption pathways or introducing NAD(P)H generation pathways [49–51]. In the following sections, metabolic engineering strategies applied to microbial production of 1,2-PDO will be reviewed. The details are depicted in Table 2.

**Table 2:** 1,2-Propandiol production in engineering bacteria

Host	Pathway involved	Substrates	Genetic modification	1,2-propanediol concentration	References
*E. coli*	Methylglyoxal pathway	Glucose	*gldA* (*E. coli*)↑/*dhaD* (*K. pneumoniae*)↑/*mgsA* (*E. coli*)↑	0.7 g/L	[52]
*E. coli*	Methylglyoxal pathway	Glucose	*mgsA* (*B. subtilis*)↑/*gldA* (*E. coli*)↑/*fucO* (*E. coli*)↑/Δ*zwf*/Δ*tpiA*/Δ*poxB*/Δ*ackA*/Δ*ubiC*	1.2 g/L	[73]
*E. coli*	Methylglyoxal pathway	Glucose	*fdh1* (*C. boidinii*)↑/*mgsA* (*B. subtilis*)↑/*gldA* (*E. coli*)↑/*fucO* (*E. coli*)↑/Δ*zwf*/Δ*tpiA*/Δ*ldhA*/Δ*gloA*/Δ*adhE*	5.13 g/L	[74]
*E. coli*	Methylglyoxal pathway	Glycerol	*mgsA* (*E. coli*)↑/*yqhD* (*E. coli*)↑/*gldA* (*E.coli*)↑/Δ*ackA*-*pta*/Δ*ldhA*/*dhaK*::*dhaKL* (*Citrobacter freundii*)	5.6 g/L	[67]
*E. coli*	Methylglyoxal pathway	Glycerol	*mgsA* (*E. coli*)/*dhak* (*E. coli*)/*gldA* (*E. coli*)/*fucO* (*E.coli*) = > BMC	11.56 mM/OD_600_	[97]
*E. coli*	Methylglyoxal pathway	Cellobiose	BGL Tfu0937 (*Thermobifida fusca* YX)↑/*mgsA* (*B. subtilis*)↑/*gldA* (*E. coli*)↑/*fucO* (*E. coli*)↑/Δ*ptsG*/Δ*tpiA*/Δ*gloA*/Δ*hchA*/Δ*ldhA*/Δ*aldA*	0.11 g/L	[49]
*E. coli*	Lactate pathway	Glucose	*MavCAR* (*Mycobacterium avium*)↑/*yahK* (*E. coli*)↑/Δ*dld*/Δ*lldD*/Δ*adhE*/Δ*frdA*/Δ*pflB*/Δ*mgsA*/Δ*aldA* or *ldhA*::*lldh* (*Pediococcus acidilactici*)	0.73 g/L *S*-1,2-PDO 0.53 g/L *R*-1,2-PDO	[99]
*E. coli*	Lactate pathway	Glucose	*pct* (*M. elsdenii*)↑/*pdup* (*S. enterica*)↑/*yahK* (*E. coli*)↑/Δ*dld*/Δ*lldD*/Δ*adhE*/Δ*frdA*/Δ*pflB*/Δ*mgsA*/Δ*aldA*/Δ*arcA* or *ldhA*::*lldh* (*Pediococcus acidilactici*)	9.3 g/L *S*-1,2-PDO 17.3 g/L *R*-1,2-PDO	[48]
*E. coli*	Lactate pathway	L-lactate	*pct* (*Clostridium propionicum DSM 1682*)↑/*pdcD* (*Yersinia enterocolitica subsp. enterocolitica 8081*)↑/*mmsB* (*Bacillus cereus ATCC 14579*)↑/*Lldh* (*Bacillus coagulans*)↑/*Zppdc* (*Zymobacter palmae*)↑/mhpF (*E. coli MG1655*)↑/Δ*lldD*/Δ*ackA-pta*/Δ*adhE*/Δ*poxB*/Δ*ldhA*/Δ*mgsA*/Δ*frdABCD*	1.04 g/L *S*-1,2-PDO	[98]
*E. coli*	Lactate pathway	l/d-lactate	*pct* (*M. elsdenii*)↑/*pdup* (*S. enterica*)↑/*yahK* (*E. coli*)↑/Δ*lldD*/Δ*dld*/Δ*ldhA*/Δ*adhE*	1.7 g/L *S*-1,2-PDO 1.5 g/L *R*-1,2-PDO	[47]
*E. coli*	Lactate pathway	Starch	*amyA* (*Streptococcus bovis*)↑/*pct* (*M. elsdenii*)↑/*pdup* (*S. enterica*)↑/*yahK* (*E. coli*)↑	0.028 g/L	[62]
*S. cerevisiae*	Methylglyoxal pathway	Glucose	*mgsA* (*E. coli*)↑/*gldA* (*E. coli*)↑/Δ*tpi1*	1.11 g/L	[80]
*S. cerevisiae*	Methylglyoxal pathway	Glycerol	*mgsA* (*E. coli*)↑/*gldA* (*E. coli*)↑/*GUT1* (*S. cerevisiae*)↑/*GUT2* (*S. cerevisiae*)↑/*gdh* (*S. cerevisiae*)↑/*GUP1* (*S. cerevisiae*)↑	2.19 g/L	[58]
*S. cerevisiae*	Methylglyoxal pathway	Glycerol	*mgsA* (*E. coli*)↑/*yqhD* (*E. coli*)↑/*gldA* (*E.coli*)↑/*Opgdh* (*O. parapolymorpha*)↑/*DAK1* (*S. cerevisiae*)↑/TPI1 (*S. cerevisiae*)↓/Δ*GUT1*	> 4 g/L	[51]
*S. cerevisiae*	Methylglyoxal pathway	Galactose	*mgsA* (*E. coli*)↑/*dhaD* (*Citrobacter freundii*)↑	0.45 g/L	[59]
*C. glutamicum*	Methylglyoxal pathway	Glucose	*mgsA* (*E. coli*)↑/*cgR_2242* (*C. glutamicum*)↑	1.83 g/L	[53]
*C. glutamicum*	Methylglyoxal pathway	Glucose	*mgsA* (*E. coli*)↑/*yqhD* (*E. coli*)↑/*gldA* (*E.coli*)↑/Δ*hdpA*/Δ*ldh*	0.343 mol/mol glucose	[13]
*C. glutamicum*	–	Pyrolysis water	*gldA* (*E.coli*)↑/Δ*pqo*/Δ*aceE*/Δ*ldhA*/Δ*mdh*	1.39 g/L	[63]
*Synechococcus elongatus PCC 7942*	Methylglyoxal pathway	CO_2_	*mgsA* (*E. coli*)↑/*yqhD* (*E. coli*)↑/*adh* (*Clostridium beijerinckii*)↑	~ 0.15 g/L	[65]
*Synechocystis sp. PCC6803*	Methylglyoxal pathway	CO_2_	*mgsA* (*E. coli*)↑/*yqhD* (*E. coli*)↑/*adh* (*Clostridium beijerinckii*)↑/Δ*glgC*	~ 1 g/L	[66]

### Enhancement of major metabolic pathways and substrate utilization range

For 1,2-PDO microbial production through the methylglyoxal pathway, three key enzymes are involved, i.e., *mgsA*, *yqhD* and *gldA*. Many methods to upregulate the expression of these three key enzymes have been implemented to increase the production of 1,2-PDO. A recombinant *E. coli* strain was constructed by Altaras and associates for synthesizing 1,2-PDO as a fermentation product of glucose, in which the expression of *mgsA* and *gldA* were upregulated simultaneously. Under the anaerobic condition of flask fermentation, compared with the original strain, 1,2-PDO production of the recombinant strain increased by 180% [52]. Enhancement of synthetic pathways involves both modification of endogenous pathways, as described in the case study above, and introduction of heterologous metabolic pathways. Niimi and Suzuki reported that introduction of *mgsA* from *E. coli* to *Corynebacterium glutamicum* increased the 1,2-PDO yield 100-fold compared with that produced by wild-type *C. glutamicum*. Furthermore, after overexpressing *mgsA* and *cgR_2242*, one of the genes annotated as AKRs that functions as a methylglyoxal reductase in the synthetic pathway, the production of 1,2-PDO doubled from 12 to 24 mM [53]. Similarly, to produce 1,2-PDO using glycerol as the main carbon source in *Saccharomyces cerevisiae*, a 1,2-PDO-producing *S. cerevisiae* was successfully metabolically engineered by combining overexpression of endogenous pathway genes with introduction of heterologous genes. Both glycerol utilization and the growth rate of the engineered strain increased after overexpressing endogenous glycerol dissimilation pathway genes, including glycerol kinase (*GUT1*) [54], glycerol 3-phosphate dehydrogenase (*GUT2*) [55], glycerol dehydrogenase (*gdh*) [56], and a glycerol transporter gene (*GUP1*) [57]. The redox balance of the strain was further improved by introducing the 1,2-PDO pathway genes *mgsA* and *gldA* from *E. coli*, and a titre of 2.19 g/L 1,2-PDO was obtained [58]. In addition, 0.45 g/L 1,2-PDO was produced from galactose after 72 h of batch fermentation by introducing the *mgsA* gene of *E. coli*-K12 MG1655 and the *dhaD* (glycerol dehydrogenase) gene of *Citrobacter freundii* in *S. cerevisiae* [59]. Both the overexpression of endogenous genes and the introduction of heterologous genes are widely used in metabolic engineering to improve the biosynthetic efficiency [60].

In addition to enhancing biosynthetic pathways, utilizing less expensive alternative material as a substrate to reduce the cost of fermentation and push the industrialization process of 1,2-PDO biosynthesis is another valuable strategy [61]. Sato et al. took the first step towards extending the substrate spectrum of 1,2-PDO biosynthesis. They successfully accomplished 1,2-PDO direct production from starch by an engineered *E*. *coli* expressing heterologous α-amylase and 1,2-PDO synthetic genes; 13 mg/L 1,2-PDO was achieved [62]. This was the first attempt to simplify the upstream saccharification process of 1,2-PDO biosynthesis. To sustain the profitability and efficiency of the conversion process in the pyrolysis of wheat straw, Lange et al. established a 1,2-PDO microbial fermentation system in pyrolysis water [63]. After introducing glycerol dehydrogenase from *E. coli* in *C. glutamicum*, a two-phase aerobic/microaerobic fed-batch process was carried out, and 18.3 ± 1.2 mM 1,2-PDO was obtained with pyrolysis water as the substrate. This result achieved the so far highest overall volumetric productivity with 1.4 ± 0.1 mmol 1,2-PDO L^−1^ h^−1^ in an engineered microbial strain, which shows the huge prospect of converting the side stream pyrolysis water to other valuable chemicals.

It is well known that reducing the amount of greenhouse gas CO_2_ emitted by industry into the environment helps mitigate the effects of global warming [64]. In this respect, the production of basic chemicals through direct fermentation of CO_2_ is a possible solution. An engineered cyanobacterium *S. elongatus* PCC 7942 produced ~ 150 mg/L 1,2-PDO, which takes in *mgsA* and *yqhD* both from *E. coli* and a second alcohol dehydrogenase (*sADH*) from *Clostridium beijerinckii* [65]. Furthermore, David al et al. achieved an ~ 1 g/L 1,2-PDO yield through optimization of cultivation conditions based on research [66]. Both these studies revealed the potential of engineered cyanobacteria to produce chemicals. In addition, direct utilization of CO_2_ dispels any concerns over competition for arable land with food crops, in contrast with biological 1,2-PDO production processes that are based on sugar or glycerol as a substrate.

### Redistribution of carbon flux

Because the 1,2-PDO biosynthetic pathways are complex, the accumulation of byproducts, which mainly include lactate, formate, acetate, succinate, pyruvate and ethanol, inevitably inhibits the production of 1,2-PDO, for example, cell growth and protein expression may be influenced by the accumulation of acetate at harmful levels [67]. With the development of genetic engineering technology in the twenty-first century, the low productivity of 1,2-PDO biosynthesis can be potentially improved using gene editing technology to redirect carbon flux [68]. Surprisingly, a substantial decrease in 1,2-PDO production was obtained with engineered *E. coli* via the methylglyoxal pathway, along with the accumulation of pyruvate and an increase in other fermentative byproducts; only the major fermentative byproduct pathway was eliminated, such as the acetate and lactate synthesis pathways. Although the engineered strain that disrupted acetate-producing pathways (acetate kinase, *ackA*; pyruvate dehydrogenase, *poxB*) [69, 70] showed lower levels of accumulated acetate (2.97 g/L) than the wild-type strain at 4 g/L, the production of 1,2-PDO was reduced, from 0.25 to 0.17 g/L, under shake flask conditions. Analogously, disruption of lactate-producing pathways (glyoxalase I, *gloA*; l-lactate dehydrogenase, *ldhA*) [71, 72] in another engineered *E. coli* strain resulted in a reduction in the 1,2-PDO titre by ~ 41%, compared with the wild-type strain, with an increase in the accumulation of other byproducts at the same time [73, 74]. Consistent with most studies, it was observed that deletion of a few key genes did not completely eliminate major byproduct production. These results all indicated that the disruption of only major byproduct pathways alone was insufficient to tap into the carbon flux for the production of 1,2-PDO. Hence, improving the accumulation of DHAP, which is considered the key precursor of the 1,2-PDO synthetic pathway, was speculated to be a potential strategy to improve 1,2-PDO production. Either the disruption of glucose 6-phosphate dehydrogenase (*zwf*) [75], leading to the activation of the pentose phosphate pathway to increase the accumulation of upstream products, or the disruption of triose phosphate isomerase (*tpiA*) [76], leading to increased glyceraldehyde 3-phosphate levels to decrease the consumption of DHAP, was successfully applied to improve 1,2-PDO production. Combining these two strategies with the deletion of genes encoding byproducts, the engineered strain generated a lower level of byproducts, with lactate at 0.14 g/L, succinate at 0.22 g/L, formate at 0.33 g/L, acetate at 0.65 g/L, and ethanol at 0.05 g/L, and more 1,2-PDO at 0.38 g/L, after 96 h, compared to 0.25 g/L from the wild-type strain. Furthermore, the galactose permease/glucokinase system (GGS) was substituted for the phosphotransferase system (PTS) to reduce phosphoenolpyruvate (PEP) consumption and carbon flow to mitigate downstream glycolysis in *E. coli*. The expression PTS-related gene *ptsG* (fused glucose-specific PTS enzymes) [77] was disrupted, and then, the GGS operon containing the *galP* (galactose permease) [78] and *glk* (glucokinase) [79] genes was introduced. As expected, 1,2-PDO was 1.57-fold more concentrated in the mutant than that in the unmodified strain (0.59 ± 0.13 g/L at 74 h) [73]. Although most of the transformations are carried out with *E. coli*, *Saccharomyces cerevisiae* is also a good choice. 1,2-PDO production from glycerol was increased 1.5-fold in *S. cerevisiae* upon deletion of the *tpi1* gene encoding glyceraldehyde 3-phosphate, which shifted the carbon flux to the DHAP side [80].

### Improving the supply of cofactors

The oxidation–reduction reaction of micro-organisms usually requires the participation of specific cofactors [81]. An insufficient supply of cofactors is often a limiting factor affecting product accumulation [82]. Therefore, genetic engineering of redox cofactors has gradually become an important metabolic engineering strategy for optimizing microbial production. In the 1,2-PDO microbial synthetic pathway, the formation of many byproducts is often accompanied by the consumption of NADH, including lactate, ethanol and succinate. Hence, it is necessary to block the synthetic pathways of all these byproducts. In addition, the overexpression of formate dehydrogenase, which shows an efficient catalytic ability to regenerate NADH from formate, is another universal strategy to increase NADH availability [83]. By introducing *Candida boidinii* formate dehydrogenase (*fdh1*), the titre of 1,2-PDO was increased by 68.57% compared to the engineered strain without an NADH regenerating system. Together with the deletion of *zwf* encoding glucose 6-phosphate dehydrogenase, *tpiA* encoding triose phosphate isomerase, *ldhA* encoding lactate dehydrogenase, *gloA* encoding glyoxalase I, and *adhE* encoding alcohol dehydrogenase (ethanol generating pathway) and the development of cell adaptation in low-phosphate formate medium, the engineered strain achieved 5.13 g/L 1,2-PDO production with a high yield of 0.48 g of 1,2-PDO/g of glucose. In addition, it has been previously suggested that the disruption of the ubiquinone biosynthesis pathway of chorismate pyruvate lyase (*ubiC*) is conducive to conserving intracellular NADH for reduction reactions. The combined effects of *ubiC* deletion with carbon flux redirection resulted in a titre of 1.2 g/L 1,2-PDO in a shake flask [73, 74].

As another commonly employed industrial strain, *S. cerevisiae* does not provide the same level of cytosolic reducing equivalents to 1,2-PDO production as the native FAD-dependent glycerol catabolic pathway. It has been recently revealed that an NAD^+^-dependent ‘DHA pathway’ successfully replaced the native pathway in *S. cerevisiae* through the heterologous expression of glycerol dehydrogenase from *Ogataea parapolymorpha* (*Opgdh*), overexpression of endogenous dihydroxyacetone kinase (*DAK1*) and deletion of endogenous glycerol kinase (*GUT1*). These modifications enabled efficient *S. cerevisiae* delivery of cytosolic NADH during 1,2-PDO microbial production. Applying strategies to increase both metabolic precursor and cofactor supplies, the modified *S. cerevisiae* strain obtained the highest titre, > 4 g/L 1,2-PDO, in yeast thus far [51]. Interestingly, NADPH-dependent alcohol dehydrogenases exhibited better reduction ability than NADH-dependent dehydrogenases in a recombined cyanobacterium producing 1,2-PDO [65]. The same result was apparent in recombined *C. glutamicum,* which proved that NADPH-dependent alcohol dehydrogenase is beneficial to anabolic metabolism [13]. Furthermore, developing genetic engineering strategies to improve the provision of NADPH, which has been proven effective in *C. glutamicum*, may be helpful to increase 1,2-PDO production. These strategies include the following: (a) overexpression of the *E. coli pntAB* genes encoding a membrane-bound transhydrogenase to leverage the electrochemical proton gradient across the cell membrane to drive the reduction of NADP^+^ upon the oxidation of NADH [84]; (b) construction of phosphoglucose isomerase (*PGI*) deletion mutants in *C. glutamicum* for redirecting carbon flux to the pentose phosphate pathway and increase the NADPH level [85]; (c) construction of a new NADPH supply channel by changing the coenzyme specificity of natural NAD^+^-dependent glyceraldehyde 3-phosphate dehydrogenase to NADP^+^ [86]; and (d) overexpression of the key enzyme NAD^+^ kinase, which converts NADP^+^ into NADPH, to increase the supply of NADPH [87]. All of these strategies may provide new solutions to the problem of an insufficient supply of cofactors in the process of microbial synthesis.

### New directions of metabolic engineering strategies

Toxic intermediates often emerge in the process of microbial synthesis, which affects not only the growth of cells but also the synthesis of the product. Hence, preventing exposure of cells to toxic intermediates cannot be overlooked [88]. The synthesis process of 1,2-PDO via the methylglyoxal pathway is often accompanied by the accumulation of toxic intermediates, such as methylglyoxal and lactaldehyde. In this case, reducing the accumulation of toxic intermediates is particularly important for the synthesis of 1,2-PDO. A technology involving scaffold strategy may be a possible solution strategy. Many signal proteins contain modular protein interaction domains that can specifically bind to other domains or short peptides. The scaffold protein with multiple protein interaction domains can interact with enzymes with polypeptide ligand tags to co-locate enzymes in metabolic pathways [89, 90]. By changing the number of domains on the protein scaffold, the relative proportions of different enzymes can be controlled. The accumulation of the toxic intermediate HMG-CoA (Hydroxymethylglutaryl-CoA) is an inevitable problem in the production of mevalonate from acetyl-CoA. Dueber et al. used protein scaffold technology to adjust the ratio of HMG-CoA production and consumption enzymes, which increased the production of the target product mevalonate by 10 times [91]. Analogously, Conrado et al. used 1,2-PDO biosynthetic enzymes to express in cells carrying DNA scaffolds containing the corresponding zinc-finger binding domains, increasing the yield of 1,2-PDO by 3.5-fold compared with that of the control without a DNA scaffold [92]. Microcompartments (bacterial microcompartments, BMCs) are another aspect of the transformation strategy of micro-organisms; BMCs can encapsulate pathway enzymes into protein shells to resolve issues of microbial instability and metabolic intermediates [93–95]. The Pdu BMC, which is a natural microcompartment, has been applied to the synthetic pathway of 1,2-PDO [96]. Lee et al. reduced the impact of toxic intermediates on cells through microcompartment technology in which the enzymes in the synthetic pathway were wrapped in a protein shell. An artificial microcompartment for synthesizing 1,2-PDO was then constructed by combining the enzymes related to synthesizing 1,2-PDO in *E. coli* with the N-terminal targeting sequence of the Pdu BMCs. Compared with that of the strain including free enzymes, the 1,2-PDO yield of the strain containing the fusion enzymes was increased by 245% [97].

In recent years, to avoid the production of toxic intermediates, an artificial and methylglyoxal-independent 1,2-PDO synthesis route was proposed and demonstrated. As a metabolic precursor, lactic acid is reduced to lactaldehyde through the joint action of propionate CoA transferase and CoA-dependent lactaldehyde dehydrogenase or the one-step action of carboxylic acid reductase, which is further reduced by alcohol dehydrogenase to 1,2-PDO (Fig. 3). In recombinant *E. coli* with the l-lactate dehydrogenase encoding gene *lldD* and the d-lactate dehydrogenase encoding gene *dld* deleted, 1,2-PDO stereoisomers were produced through the catalysis of a propionate CoA transferase, encoded by *pct* gene from *Megasphaera elsdenii*, a CoA-dependent aldehyde dehydrogenase, encoded by *pduP* from *Salmonella enterica*, and an alcohol dehydrogenase, encoded by *yahK* from *E. coli*. The modified strain produced 1.5 g/L *R*-1,2-PDO and 1.7 g/L *S*-1,2-PDO from d-lactic acid and l-lactic acid under shake flask conditions, respectively [47]. Furthermore, 17.3 g/L *R*-1,2-PDO and 9.3 g/L *S*-1,2-PDO were biosynthesized from glucose under fermentation-controlled conditions, while 97.5% *ee* (*R*) and 99.3% *ee* (*S*) of the optical purity was obtained. These are the highest titres of 1,2-PDO microbial synthesis obtained thus far [48]. The same pathway was constructed in *E. coli* to convert l-lactic acid to *S*-1,2-PDO with the deletion of genes related to the methylglyoxal bypass pathway. *S*-1,2-PDO (13.7 mM) was produced from glucose by redistribution of carbon flux and introduction of a cofactor regeneration system [98]. In addition, Kramer et al. synthesized 1,2-PDO from lactic acid by directly overexpressing the carboxylic acid reductase gene *MavCAR* from *Mycobacterium avium* and the alcohol dehydrogenase-related gene *yahK* from *E. coli*. *R*-1,2-PDO accumulated at 7.0 mM with a molar yield of 1.0%, while the *S*-isomer was produced from glucose at 9.6 mM with a molar yield of 1.4% [99]. Compared to the CoA-dependent 1,2-PDO synthesis pathway, this route is simpler and more convenient, and only requires a one-step reaction, from which lactic acid produces lactaldehyde. With the development of more efficient enzymes, novel synthetic pathways and new metabolic engineering strategies, the field of microbial synthesis will inevitably expand with new vitality.

## Future perspectives

As described at the beginning of this review, 1,2-PDO has immense potential in the global market but an immature industrial microbial synthetic process. Current 1,2-PDO biosynthesis routes based on biomaterials, including glycerol, starch and cellulose, and its microbial conversion reaction do not fully correct the limited substrate spectrum or relatively low yields nor produce economic benefits. However, the recent construction of several complete biosynthetic routes in different strains is coming to the forefront, especially for the production of 1,2-PDO stereoisomers from glucose in *E. coli,* which is instructive to the development of effective 1,2-PDO microbial production. First, novel inexpensive substrates should be developed to reduce costs of 1,2-PDO commercial manufacturing. Using genetic engineering technology, it is possible to utilize inexpensive substrates for 1,2-PDO microbial biotransformation, such as glycerol, cellulose, CO_2_, etc. Second, a series of bioengineering strategies need to be applied to improving the yields and titre of 1,2-PDO. Mining the key high-efficiency enzymes used in the reduction reaction is an effective method to increase the production of 1,2-PDO. Li et al. exploited NADPH-specific secondary alcohol dehydrogenases, which increased the production from 22 to 150 mg/L [65]. Hence, obtaining a highly active enzyme with good thermostability and desired substrate specificity is significant for increasing production. The structural biology analysis of different key rate-limiting enzymes has been helpful in understanding their catalytic mechanism and substrate specificity; hence, enzymes can be directly modified through protein engineering methods such as rational or semirational design to achieve higher yields of target products [100]. In addition, rate-limiting enzyme obtained by screening biological databases using computational techniques [101] and their functionalities predicted using BLAST searches are the primary virtual means employed for key enzyme discovery in 1,2-PDO biosynthesis [102]. Moreover, a previous study revealed that methylglyoxal and lactaldehyde have toxic inhibitory effects on strains to suppress cell growth. Therefore, solving the problem of toxic intermediate is vital for the improvement of production. Recent research has mostly focused on the reduction of toxic intermediates, while another neglected potential strategy is the enhancement of the tolerance of chassis cells to toxic intermediates [103]. This strategy is conducive to building more robust chassis cells through the combination of adaptive laboratory evolution [104], high-throughput screening [105] and leveraging the toxic mechanisms of related intermediates. Furthermore, different chassis micro-organisms have different advantages, and nonmodel organisms have received extensive attention from researchers because of their specific metabolic networks [106]. With the rapid development and improvement of synthetic biological tools, for example CRISPR/Cas9 [107], gene editing of micro-organisms with different chassis cells, and even unmodelled organisms, has gradually become possible [108]. Hence, in-depth research on chassis cells will hopefully establish more suitable 1,2-PDO synthetic chassis cells and accelerate the industrialization of 1,2-PDO biosynthesis. Finally, it is necessary to develop the downstream product transformation of a variety of high value-added chemicals to further increase the value of 1,2-PDO and broaden the application field.

## Conclusion

Now the process of 1,2-PDO by biosynthesis is commercially infeasible because of the expensive cost. One of the most important causes of this is the low efficiency of enzymes involved in the 1,2-PDO microbial synthetic pathways. Hence, it is essential to look for high-efficiency enzymes, which are suitable for the production of 1,2-PDO. In addition, although the micro-organisms and biosynthetic pathways for microbial production of 1,2-PDO are relatively abundant, it is the primary task to select strains and pathways suitable for industrial production. In this review, we summarized a variety of micro-organisms and 1,2-PDO biosynthetic pathways that have the potential to be applied to industrialized manufacturing. The applicable environment of different strains varies a lot, so it is important to select the corresponding strains according to the environment. For instance, *C. thermosaccharolyticum* prefers the high-temperature environment, while *Lactobacillus buchneri* prefers the acidic environment. Furthermore, the three known 1,2-PDO biosynthetic pathways also have their pros and cons. Although the deoxyhexose pathway is convenient to obtain strains, the cost of the substrate is relatively high; the research on the methylglyoxal pathway is relatively detailed, but the problem of toxic intermediates is difficult to solve; the lactic acid pathway is relatively simple and fast, but the details still need to be further explored. We also summarized strategies for enhancing the synthesis of 1,2-PDO, such as the overexpression and introduction of key genes to improve the synthesis efficiency of 1,2-PDO, the knockout of related genes in the byproduct synthesis pathways to reduce the accumulation of byproducts, and the construction of cofactors circulation system to supply sufficient NAD(P)H for a series of reduction reactions. With the optimization strategies summarized in this review, it is promising to use micro-organisms to produce 1,2-PDO with relatively high yields.

Collectively, further studies on the biological synthesis of 1,2-PDO will contribute to alleviating petroleum shortages. A series of microbial cell factories producing 1,2-PDO have recently been constructed, while efficient microbial synthetic methods of 1,2-PDO are still lacking, and more exploration in synthetic biology is needed. In summary, we can expect that further metabolic engineering strategies will lead to a highly efficient 1,2-PDO production process in recombinant microbes.

## Data Availability

Not applicable.

## Abbreviations

1,2-PDO: 1,2-Propanediol
CAGR: Compound Annual Growth Rate
BMC: Bacterial microcompartment
DHAP: dihydroxyacetone phosphate
GGS: Galactose permease/glucokinase system
PTS: Phosphotransferase system
PEP: Phosphoenolpyruvate
HMG-CoA: Hydroxymethylglutaryl-CoA
*ackA*: Acetate kinase
*adhE*: Alcohol dehydrogenase
*DAK1*: Dihydroxyacetone kinase
*dhaD*: Glycerol dehydrogenase
*dld*: d-Lactate dehydrogenase
*fdh1*: Formate dehydrogenase
*fucA*: l-Fuculose-1-phosphate aldolase
*fucI*: l-Fucose isomerase
*fucK*: l-Fuculokinase
*fucO*: Propanediol oxidoreductase
*galP*: Galactose permease
*gdh*: Glycerol dehydrogenase
*gldA*: Glycerol dehydrogenase
*glk*: Glucokinase
*gloA*: Glyoxalase I
*GUP1*: A glycerol transporter gene
*GUT1*: Glycerol kinase
*GUT2*: Glycerol 3-phosphate dehydrogenase
*ldhA*/*lldD*: l-Lactate dehydrogenase
MavCAR: Carboxylic acid reductase from *Mycobacterium avium*
*mgsA*: Methylglyoxal synthase
*Opgdh*: Glycerol dehydrogenase from *Ogataea parapolymorpha*
*pct*: Propionate CoA-transferase
*pduP*: Propanal dehydrogenase
*PGI*: Phosphoglucose isomerase
*pntAB*: Membrane-bound transhydrogenase
*poxB*: Pyruvate dehydrogenase
*ptsG*: Fused glucose-specific PTS enzymes
*rhaA*: l-Rhamnose isomerase
*rhaB*: Rhamnulokinase
*rhaD*: Rhamnulose-1-phosphate aldolase
*sADH*: Secondary alcohol dehydrogenase
*tpiA*/*1*: Triose phosphate isomerase
*ubiC*: Ubiquinone biosynthesis pathway-chorismate pyruvate lyase
*yqhD*: Alcohol dehydrogenase
*yahK*: Lactaldehyde reductase
*zwf*: Glucose-6-phosphate dehydrogenase
